# Single Patient Multiple Explosions: A Case Report on Exploding Head Syndrome

**DOI:** 10.7759/cureus.44437

**Published:** 2023-08-31

**Authors:** Wasef Alkhateeb, Abhinaya Krishnaraj, Vishal Saini

**Affiliations:** 1 Department of Internal Medicine, Central Michigan University College of Medicine, Saginaw, USA

**Keywords:** loud noise, sleep disorder, sleep medicine, parasomnia, exploding head syndrome

## Abstract

Exploding head syndrome (EHS) is an uncommon sleeping disorder that is described by patients as a loud noise occurring while transitioning into and out of sleep. It is not accompanied by a headache but causes a sense of fright. We describe the case of a 58-year-old female patient, presenting with a total of 11 events of EHS occurring at bedtime. Events shared some features but also had unique characteristics. The workup was negative, making a diagnosis of EHS more likely. CPAP was recommended for a newly diagnosed obstructive sleep apnea, but despite non-compliance with the treatment plan, the patient had a complete resolution of her symptoms. Exploding head syndrome is an underreported parasomnia reported in the literature. Our case report demonstrates that a single patient could exhibit different features in displaying EHS, which was shown by changes in the noise description, pattern, and accompanying jerk movement. It also hints at patient education and reassurance as a possible treatment plan.

## Introduction

Parasomnias are sleep disorders that involve unusual and undesirable physical events that disturb sleep [1]. These disorders are split into REM and non-REM-occurring parasomnias [1]. One of the uncommon parasomnias described in the literature is exploding head syndrome (EHS) [1]. 

EHS has been described by patients who experienced it as loud noise originating from within the head [2]. Noises described were explosions, gunshots, fireworks, lightning, door slamming, roars, waves crashing against rocks, loud voices, a terrific bang on a tin tray, or sounds of electrical buzzing [2]. Flashing lights were also reported to accompany these episodes in some cases. Headaches were not reported as a result of or during the event. However, patients did report being frightened by it [2]. 

Exploding head syndrome was first written in literature by neurologist Silas Weir Mitchell in 1890, and since then, there have been cases reported periodically. It was officially placed in the International Classification of Sleep Disorders in 2005 [3]. However, there have been very few clinical reports of this condition, and a literature review in 2014 yielded 76 cases of this syndrome with specific criteria [4].

We present the case of a female patient complaining of multiple events of EHS with varying event characteristics. We discuss the diagnosis, workup, and management of EHS. 

## Case presentation

A 58-year-old female patient with a controlled medical history of hypertension, diabetes, and psychiatric conditions, including anxiety, depression, bipolar, and PTSD. She presented to her primary care provider with a complaint of multiple events of loud noises occurring as she was falling asleep. 

The patient described 11 events over the past year occurring almost three to four weeks apart. The first event was described as Cymbals banging together; it occurred as the patient was dozing off in the seated position. She described the noise within her head going from the right side to the left side of her head, accompanied by a head and neck jerk in the direction of the noise. The event lasted less than a second. She didn’t feel any pain from the event but reported being frightened, thinking she might be hallucinating. One month later, the patient described having had an identical event to the first attack.

Over the next five months, she stated having one attack a month. The events were very similar in the sense of timing, noise description, head jerk, and duration. But it occurred while that patient was lying on her left side, as opposed to her first two attacks, where she was in the seated position.

From that point on, the patient noticed that the events had changed in nature and were occurring more frequently. The eighth event was described as four cymbal noises back-to-back, all four occurring with the head jerks. Similar to previous events, the noises also originated from the right side of the head towards the left. Three weeks later, the patient had an identical event, with another four noises instead of one.

The next two events occurred one month later, three weeks apart. Interestingly, the patient described new characteristics of the events. This time, instead of cymbals, the patient described hearing bomb sounds. Instead of origination from right to left, the noise was noted to start in the occipital region and travel anteriorly to the frontal region. The jerk followed the direction of the noise, with the event causing neck flexion rather than a side-to-side jerk seen in previous events.

On questioning, no identifiable trigger event that might have preceded the events was identified. The patient noted a stressful event that occurred three years before the first episode. All events occurred as the patient fell asleep and was not fully awake, lasting less than a second each time. Events always cause fright. She denied headaches at the time of the event, no weakness or numbness, and no reported loss of bowel or bladder control. 

When asked about her sleep, the patient did state that she stays up late most days but has consistent sleeping habits, with no recent changes in her sleep schedule. She sleeps around five to six hours every night. She is able to remain asleep throughout the night. She feels refreshed upon waking up in the morning and doesn’t take naps during the day.

Her past medical history is significant for hypertension controlled on amlodipine, lisinopril, and hydrochlorothiazide. Type 2 diabetes mellitus is controlled with metformin and diet. Multiple psychiatric conditions, chronically on Escitalopram, Lamotrigine, Lorazepam, Ziprasidone, and Prazosin, which were added three years before the first event. The patient denied any recent medication changes and denied any missed doses. 

Regarding her social history, her husband passed away three years ago, which was a significant stressor for her. She’s an ex-smoker who quit more than 20 years ago; denies alcohol use but admits to using cannabinoids daily since she was a teenager. She’s independent in her daily activities and lives alone.

A physical examination showed normal vital signs. BMI of 33. Mallampati score is II. The neurological exam, including cranial nerves, motor, sensory, cerebellar, and cognitive function, was unremarkable. She noted that she lost over 45 pounds intentionally. PHQ-9 score 3, GAD-7 score 12, indicating moderate anxiety.

The workup included a complete blood count with differential and a basic metabolic panel, which were normal. Iron, vitamin B12, and vitamin D levels were normal. The patient underwent non-contrast head computed tomography (CT), showing mild bifrontal cortical atrophy, no acute infarct, intracranial hemorrhage, edema, or mass, and no midline shift.

**Figure 1:**
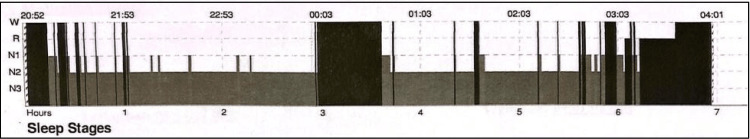
Polysomnography The figure demonstrates the stages of sleep seen during our patient's polysomnography.

**Figure 2:**
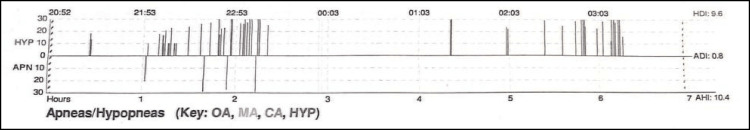
Polysomnography, apnea-hypopnea section The figure demonstrates episodes of apnea and hypopnea event recorded during our patient's polysomnography.

Regarding her sleep assessment, she had an Epworth sleepiness score of 1/24 and a STOP-BANG score of 5/8. Overnight polysomnography (PSG) (Figure 1) resulted in a total sleep time of 5 hours and 14 minutes, with a sleep efficiency of 86.2%. Sleep latency of 12.5 minutes. The patient spent most of her sleep in Stage 2 at 68.1%, with REM sleep at 15.6%. The apnea-hypopnea index (AHI) was noted to be 10.37, qualifying for an obstructive sleep apnea (OSA) diagnosis (Figure 2). 

The lowest O2 saturation was 85%. Periodic Limb Movement Disorder index (PLMS) (8.3) is not enough for a PLMS diagnosis. The electroencephalogram (EEG) portion of the PSG didn’t show any epileptiform activities. The patient didn’t report any loud noises at the time of the PSG.

Auto-CPAP therapy was recommended, and she was assured that this is a benign condition. On follow-up appointments, she stated that she had not been using the CPAP machine as instructed. However, she reports having no more additional episodes or loud noises during sleep. 

## Discussion

According to the International Classification of Sleep Disorders (ICSD-3), diagnostic criteria for EHS include the following: sudden, loud sound in the head while transitioning into or out of sleep; abrupt, frightening arousal following the event; and no significant pain associated with the experience [3]. Our patient indeed meets all three criteria for EHS.

EHS was first introduced by Silas Weir Mitchell in 1890 and referred to as “sensory shock”, but the term explosive head syndrome was first used by J. M. Pearce in 1989 [5]. After a study of 50 patients described having explosive noises in their heads during sleep. Gender differences are still unclear, but EHS seems to affect females more than males, with a ratio of 1.5:1.0 [6], with a median age in the fifth decade of life, but cases have been reported in patients as young as 10 years old [2]. It is thought to occur when transitioning into and out of sleep [7]. One case report demonstrated that EHS occurred during the transition from awake to non-REM sleep stage one and from REM to awake using polysomnography [7]. 

When it comes to etiology, there is no clear evidence of why or how this condition occurs. Proposed etiologies include ear dysfunction, temporal lobe seizures, medication withdrawal, mainly benzodiazepines, selective serotonin reuptake inhibitors, and finally, brainstem neuronal dysfunction during sleep transitions [8]. One study hypothesized calcium channel dysfunction after some treatment responses to nifedipine and topiramate [6]. Possible contributing factors identified in our case are underlying OSA and multiple psychiatric conditions. Although she is on multiple medications suggested to cause EHS upon withdrawal, none of which were abruptly discontinued or had dose adjustments. 

While the inheritance and family history of EHS remain to be studied, it was hinted at in a case report by Palikh et al. They report a case of a female patient with EHS, with similar events noted in the patient’s mother and daughter [9].

Sleeping position could also be a factor in EHS. Prizada et al. describe three patients who experienced EHS more frequently when sleeping in the supine position [10], and changing their sleeping position decreased the frequency of EHS. As opposed to our patient, who experienced episodes of EHS in various sleeping positions.

The differential diagnosis for our case included hallucination disorder, cerebrovascular event, and seizure disorder. The lack of pain is a crucial feature of EHS, ruling out other conditions, most importantly subarachnoid hemorrhage. The lack of a post-ictal state helps differentiate EHS from seizure disorders, coupled with a normal EEG. A head CT enabled the ruling out of structure abnormalities, including brain tumors and ischemic or hemorrhagic strokes. 

Treating EHS is not warranted, as various studies have described the disease as benign, and reassurance alone reduces anxiety [2]. One study described successful treatment with the tricyclic antidepressants’ clomipramine and slow release nifedipine, which was noted to achieve complete remission from EHS. Although topiramate didn’t result in remission, it was stated that it decreased the intensity of the noise [8,9]. Both Frese et al. and Prizada et al. utilized amitriptyline in their cases with reasonable success, with many patients reporting significant improvement in their symptoms [4,10]. However, it is also worth mentioning that they also had success with education and sleep hygiene. Nakayama et al. expanded on that in their study, highlighting that treating underlying disorders may result in the improvement and even resolution of EHS. They described one patient with sleep apnea whose EHS completely resolved after the introduction of an oral appliance, and another with baseline depression reported resolution after ECT treatment for depression [7]. As with our case, the patient reported a resolution of episodes despite not being compliant with the CPAP machine, hinting at reassurance and education being an effective approach. 

Although it is a benign condition, it is accompanied by a sense of fright, as was also reported by our patient. Kaneko et al. describe a case where EHS caused significant fear and distress, resulting in multiple panic attacks. This necessitated the initiation of treatment, resulting in the gradual improvement of reported episodes [11].

## Conclusions

Exploding head syndrome is an underappreciated parasomnia described in the literature. EHS presents with a loud noise, mainly when transitioning into and out of sleep. Our case report demonstrates that a single patient could exhibit different features in displaying EHS, which was shown by changes in the noise description from cymbals to bomb noises and accompanying jerk movement going from side to side to back to front. When it comes to treatment, addressing the underlying disorder can result in resolution, in addition to reassuring the patient about the benignity of the condition, education, and sleep hygiene. If that proves ineffective, multiple pharmacological options have been used in the literature with reasonable success.
